# Experimental Setup Employed in the Operating Room Based on Virtual and Mixed Reality: Analysis of Pros and Cons in Open Abdomen Surgery

**DOI:** 10.1155/2020/8851964

**Published:** 2020-08-06

**Authors:** R. Galati, M. Simone, G. Barile, R. De Luca, C. Cartanese, G. Grassi

**Affiliations:** ^1^IRCCS Istituto Tumori “Giovanni Paolo II”, Department of Oncology Surgery, Bari, Italy; ^2^Dipartimento Ingegneria Innovazione, Università del Salento, Lecce, Italy

## Abstract

Currently, surgeons in operating rooms are forced to focus their attention both on the patient's body and on flat low-quality surgical monitors, in order to get all the information needed to successfully complete surgeries. The way the data are displayed leads to disturbances of the surgeon's visuals, which may affect his performances, besides the fact that other members of the surgical team do not have proper visual tools able to aid him. The idea underlying this paper is to exploit mixed reality to support surgeons during surgical procedures. In particular, the proposed experimental setup, employed in the operating room, is based on an architecture that put together the Microsoft HoloLens, a Digital Imaging and Communications in Medicine (DICOM) player and a mixed reality visualization tool (i.e., Spectator View) developed by using the Mixed Reality Toolkit in Unity with Windows 10 SDK. The suggested approach enables visual information on the patient's body as well as information on the results of medical screenings to be visualized on the surgeon's headsets. Additionally, the architecture enables any data and details to be shared by the team members or by external users during surgical operations. The paper analyses in detail advantages and drawbacks that the surgeons have found when they wore the Microsoft HoloLens headset during all the ten open abdomen surgeries conducted at the IRCCS Hospital “Giovanni Paolo II” in the city of Bari (Italy). A survey based on Likert scale demonstrates how the use of the suggested tools can increase the execution speed by allowing multitasking procedures, i.e., by checking medical images at high resolution without leaving the operating table and the patient. On the other hand, the survey also reveals an increase in the physical stress and reduced comfort due to the weight of the Microsoft HoloLens device, along with drawbacks due to the battery autonomy. Additionally, the survey seems to encourage the use of DICOM Viewer and Spectator View both for surgical education and for improving surgery outcomes. Note that the real use of the conceived platform in the operating room represents a remarkable feature of this paper, since most if not all the studies conducted so far in literature exploit mixed reality only in simulated environments and not in real operating rooms. In conclusion, the study clearly highlights that, despite the challenges required in the forthcoming years to improve the current technology, mixed reality represents a promising technique that will soon enter the operating rooms to support surgeons during surgical procedures in many hospitals across the world.

## 1. Introduction

During the last decades, the advances in electronics, mechatronics, and computer vision have been used to design very powerful and accurate microcameras, smaller and faster processors, and real-time data streaming. These devices have led to remarkable improvements in diagnostic imaging systems such as mammography, computerized tomography, and magnetic resonance imaging [1]. Surely, these tools have revolutionized the way physicians use imaging to perform procedures, particularly during emergencies when surgeons need as much information as possible about the patient in order to successfully complete the procedure.

Despite all these innovations, it is worth noting that while imaging has radically evolved, the way images are displayed is basically the same as in the past [2]. Usually, surgeons are forced to focus their attention both on their patients and on medical-grade flat surgical monitors in order to get all the information needed to avoid adverse events and, consequently, correctly finish up the surgery with an appreciable outcome for the patient. Further, medical images are not displayed from the perspective of the viewer, but rather from that of the imaging device: surgeons have to use their experience and imagination to understand and mentally project the images into the patient's body while they are doing procedures [3]. Finally, different types of visual data are displayed separately, so doctors have to focus additional attention to mentally fusing multiple image types (such as abdomen RMI and CT colonography) into a coherent representation of the patient. Note that acquiring this skill takes years of training.

Nonetheless, it seems quite unreasonable that surgeons have to look away from the surgical site in order to look at the monitor, especially during high-risk procedures that are associated with significant inpatient mortality [4]. Thus, surgeons need specific visual requirements, which are very different from those of all the other physicians. These requirements include good acuity for fine detail at close range, good depth perception, and good color vision. For this reason, surgeons need to observe more scopes or monitors during surgeries.

Few years ago, the concept of mixed reality (MR) has been introduced [5]. It represents the merging of real and virtual worlds to produce new environments and visualizations, where physical and digital objects co-exist and interact in real time [5]. Mixed reality has started entering the operating rooms in many hospitals around the world, with the aim to support surgeons and operating team. It represents a key technology that has the potential to solve all of the aforementioned problems occurring in the operating rooms [6, 7]. The use of applications based on mixed reality enables medical professionals to recreate real-world images of anatomical structures virtually while they wear specific binocular head-mounted display. The augmented information can then be projected over the patient's body in real-time and can be overlaid on the field of view of the surgeon. Using such technology, surgeons do not have to take their eyes off the patient for looking at different displays when they need to gather and interpret medical information [8]. Moreover, the possibility to use mixed reality features allows the surgeons to anchor virtual objects to the real world and interact with them. This provides a very powerful tool for better training and improving education on surgical tactics and methods [9].

One of the most popular mixed reality headsets is the Microsoft HoloLens. So far, different uses of Microsoft HoloLens have been reported in literature. Referring to training applications, in [10], a system for laparoscopy training that is enhanced by Microsoft HoloLens has been described. In particular, the system in [10] is used to perform peg-transfer training tasks via Microsoft HoloLens. In [11], suturing training has been investigated using Microsoft HoloLens. In particular, in [11], a training module for basic suturing training has been developed by combining video instruction and voice commands with the Microsoft HoloLens software. In [12], anatomy training has been proposed using a HoloLens-based augmented reality system. Referring to navigation, in [13], Microsoft HoloLens has been used for navigation in endovascular aortic repair, with the aim of reducing side effects such as radiation exposure and contrast agent administration. In [13], a novel multicriteria evaluation model for HoloLens-based mixed reality surgical navigation system is illustrated. The model, which includes factors such as comfort and safety, is performed in an actual clinical application [13]. Referring to visualization, in [14], a framework for 3D holographic visualization of myocardial scar (imaged using late gadolinium enhancement) has been developed based on Microsoft HoloLens. Such visualization of the complex 3D architecture of myocardial scar via Microsoft HoloLens has improved guidance of radiofrequency ablation in the treatment of ventricular tachycardia [14]. Referring to interactive registration, in [15], Microsoft HoloLens has been used in 3D thoracoscopy. Namely, interactive registration may help the surgeon when one or several trocars must be inserted between the patient's ribs [15]. Finally, an approach for advancing telemedicine using the Microsoft HoloLens has been illustrated in [16].

Based on previous considerations, the purpose of this study is to focus on the effectiveness of Microsoft HoloLens as a tool for aiding one or more members of the surgical team during abdominal surgeries. The aim is to evaluate the technical feasibility of introducing this kind of technology in open surgery in the operating rooms. The paper is organized as follow.  Section 2 briefly illustrates the hardware and the equipment used for this research work, whereas Section 3 provides a description of the experimental setup employed in the operating room. It enables visual information on the patient's body and on the results of medical screenings to be visualized on the surgeon's headsets, as well as any data and details to be shared by the team's members or by external users during surgical operations.  Section 4 presents a discussion on the advantages and drawbacks that the surgeons have found when they wore the Microsoft HoloLens during all the ten open abdomen surgeries conducted at the IRCCS Hospital “Giovanni Paolo II” in the city of Bari (Italy). These surgeries, carried out with the Microsoft HoloLens, represent a remarkable feature of this manuscript, since most if not all studies conducted in literature so far exploit mixed reality only in simulated environments [17] and not in real operating rooms. Finally, by using a Likert-like scale, Section 5 presents the results of questionnaires that have been given to the team's members during surgeries conducted when wearing Microsoft HoloLens.

## 2. Materials and Equipment

The aim of this section is to briefly describe the technical specifications of the Microsoft HoloLens. This will help the reader, in the following, to better understand advantages and drawbacks when adopting this device in open abdomen surgery. Microsoft HoloLens is a mixed reality headset able to display virtual additions in context to the real environment on the user's field of view. The electronic unity that calculates the visual information and the mixed reality objects is stand-alone and fully integrated into the HoloLens hardware. Thus, there is no need for extra computers or devices. Information is projected on specific see-through lenses based on holographic waveguide in front of the user's eye [18]. The HoloLens hardware includes an inertial measurement unit, which integrates an accelerometer, a gyroscope, and a magnetometer to detect the head movements in the three-dimensional space. The hardware also includes a pair of short and long-throw infrared illuminators, an energy-efficient depth camera with a 120° × 120° angle of view, a 2.4-megapixel photographic video camera, an array made up by four microphones, and an ambient light sensor [18].

**Figure 1:**
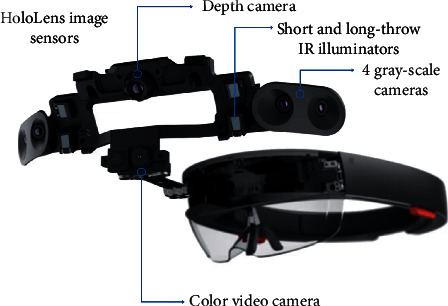
HoloLens hardware and details.

More specifically, Microsoft HoloLens is equipped with four gray-scale environment tracking cameras and a depth camera to sense its environment and capture gestures of the user. An image illustrating some details is reported in Figure 1. Moreover, two of the gray-scale cameras are configured as a stereo mode, in order to capture the area in front of the headset so that the absolute depth of tracked visual features can be determined through triangulation. The remaining two additional gray-scale cameras are used to provide a wider field of view and to keep track of features [18]. It is worth saying that these synchronized global-shutter cameras are significantly more light-sensitive than the color camera. Consequently, they can be used to capture images at a rate of up to 30 frames per second (FPS). The color video camera placed in the front of the device can provide a horizontal field of view of 67° with a maximum resolution of 1344 × 756 px when configured to work with large FOV video mode with overscan or at 1280 × 720 px with a field of view of 45° when used in standard mode.

The depth camera uses active infrared illumination to determine depth through time-of-flight [19]. The camera can operate in two modes. In particular, the first mode enables high-frequency near-depth sensing at 30 fps and is commonly used for hand tracking. The second mode is used for lower-frequency far-depth sensing at maximum 5 fps and is usually adopted by spatial mapping. In addition to depth information, this camera also delivers actively illuminated IR images that can be valuable on their own because they are illuminated from the device itself and are reasonably unaffected by ambient light [19].

In addition to an Intel Cherry Trail SoC containing the CPU and GPU, HoloLens features a custom-made Microsoft Holographic Processing Unit (HPU), which is a co-processor manufactured specifically for the HoloLens by Microsoft. The SoC and the HPU each have 1 GB LPDDR3 and share 8 MB SRAM, with the SoC also controlling 64 GB eMMC and running the Windows 10 operating system. The HPU uses 28 custom DSPs from Tensilica to process and integrate data from the sensors, handling tasks such as spatial mapping, gesture recognition, and voice/speech recognition. The hardware is powered by an internal rechargeable 16.5Wh battery and offers an average autonomy of maximum 2 or 3 hours of active use or a maximum of 2 weeks when in standby mode. To maximize the battery life and duration, HoloLens works with IEEE 802.11ac Wi-Fi and Bluetooth 4.1 Low Energy (LE) wireless connectivity [19].

## 3. Description of the Experimental Setup

This section describes our experimental setup, i.e., all the operations (hardware and software settings) required in order to allow our surgeons to effectively use Microsoft HoloLens in open abdomen surgery. To achieve this objective, it is necessary to equip one or more members of the surgical team with the Microsoft headset, so that they can visualize information about the results of medical screenings, such as radiography, blood tests, and magnetic resonance imaging. Furthermore, it is necessary to add visual information on the patient's body by using mixed reality tools. Finally, it is necessary to share information with other professionals, this being particularly useful for training, remote tutoring, and for receiving external advice from other physicians [20].

### 3.1. Management of Medical Imaging Information

**Figure 2:**
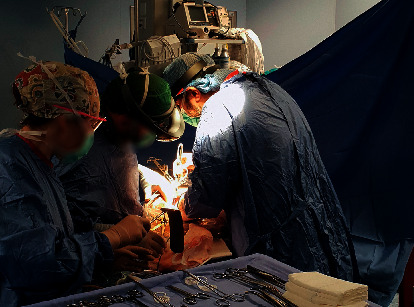
Microsoft HoloLens used during an open multivisceral resection surgery for advanced gastric adenocarcinoma with infiltration of nearby organs.

In our experimental setup, the open-source Mixed Reality ToolKit (MRTK) has been used to implement features based on mixed reality functions. This allows users to place objects, notes, or images over the real scene. MRTK is a Microsoft-driven project that provides a set of components and features used to accelerate the development of mixed reality applications in Unity [21]. Basically, the toolkit provides the building blocks for Unity development on HoloLens. Moreover, the toolkit enables rapid prototyping via in-editor simulation, which allows the user to immediately see any change [22]. By virtue of this toolkit, it is possible to place and manipulate objects in the three-dimensional space. Moreover, it is possible to track the head movements in order to autoscroll text or fluently zoom into focused content. These two functions have been used to facilitate the surgeon during the navigation among media contents or to share specific information by adding a text or a note on the real world. In order to correctly develop applications with the MRTK toolkit, Visual Studio 2019 has been used on Microsoft Windows 10 with Unity 2019 and Windows 10 SDK. The MRTK foundation package has been used as core system, whereas the scene system contained in MRTK/Examples/Experimental/Demos/ExamplesHub/Scenes/ has been used to load the scene and the content necessary to build the controls (i.e., visual buttons) or the visual tips employed for user interaction. Additionally, since Digital Imaging and Communications in Medicine (DICOM) is the widely used standard for the communication and management of medical imaging information [23], the open-source DICOM_viewer, released by the University of Auckland, has been used as starting point to implement a DICOM player, making it available on all the HoloLens devices used by each surgical team's member. The DICOM player, which is based on the Unity Game Engine, has been developed in order to enumerate all the DICOM images needed for each surgery procedure. The images have been stored in their original format in a specific folder (used as a DICOM Database) and have been shared on the main server. When requested, each image file has been converted in a STL format in order to prepare it for a three-dimensional representation via the open-source InVesalius software. This software uses VTK library to implement the direct volume rendering, with the objective to provide a scientific visualization of the medical imaging data, in addition to other projection techniques such as MIP and MIDA. Since the Unity Engine does not natively support STL file format, an additional conversion from STL to OBJ has been performed by using a C++ 3D file converter library (named jrbedard). Finally, the obtained 3D object has been added to the current scene. By using the DICOM viewer, the surgeon can load image into the real scene and perform some manipulations like rotation, segmentation, and scaling to adjust its location and size according to his needs. Finally, the open-source Spectator View toolkit has been used for this study to develop the most important feature, i.e., the possibility to share all the information generated by one of the multiple HoloLens devices with other external devices, such as smartphones, laptops, and so on. Specifically, Spectator View is a multidevice experience that allows HoloLens-based applications to be viewed by additional devices at their own viewpoints. It offers functionality for unidirectional content synchronization (state synchronization) and leverages spatial coordinates for scene alignment (spatial alignment). In our setup, the Android devices track their locations in the world relative to their local application origin by using ARCore, which supports devices running Android 7.0 or later. Each device establishes a connection to a socket available on the HoloLens device worn by the surgeon qualified to share the scene information. In order to handle and exchange localization information between the HoloLens and the Android devices, the spatial alignment component is used to provide abstractions for localization of the mixed reality content within the real world. The architecture of the spatial alignment component in the Unity includes several tools for aligning and keeping virtual objects, which are aligned with the physical world both for the HoloLens and the mobile devices. Each Android device applies a transform to its local camera (based on the location information generated by the spatial alignment component) in order to locate the perceived application origin on the mobile device in the same physical position as the local application origin on the HoloLens device. By following the Microsoft documentation, the “STATESYNC TEXTMESHPRO preprocessor” directive has been added to our Unity application. Moreover, all the assets in the project have been assigned to unique identifiers to force the content in the user's application scene to be updated dynamically in the current scene. A software runs on the external devices (that need to be connected to the augmented world) and tracks their locations in the world relative to its local application origin. After this operation, both the local device and the HoloLens device locate a shared spatial coordinate relative to their local application origins. The mobile device applies a transform to its camera based on the location of this spatial coordinate in the two local application spaces. After applying this transform, the perceived application origin on the mobile device is located in the same physical position as the local application origin on the HoloLens device. The HoloLens begins sending scene information to the mobile device and then it updates its local application content to reflect what is being observed on the HoloLens device. The Spectator View feature enables all the team members to share the surgeon's point of view, with the aim to speed up the medical routines and better support him. This can also improve training for medical doctors because they can observe the real scene with all the augmented information placed by the surgeon, with the aim to better explain and clarify certain procedures and methods. DICOM player and Spectator View have been used in all the ten open abdomen surgeries conducted at the IRCCS Hospital “Giovanni Paolo II” in Bari (Italy). While DICOM player has been used to visualize patient's data information both in 2D and in 3D (when these data were available before an intervention), the Spectator View turned out to be particularly useful for surgical telementoring and medical training, by virtue of the possibility to add virtual tooltips on the patient's body to highlight regions of interest. Note that these features are very useful in almost all the surgical interventions. This is because they provide fast tools to visualize results from medical examinations, clinical situations, and notes about complex procedures as well as to underline relevant aspects to remote users. As an example of the proposed experimental setup, Figure 2 shows two surgeons wearing Microsoft HoloLens during an open multivisceral resection surgery for advanced gastric adenocarcinoma with infiltration of nearby organs. The picture was taken in October 2019 at the IRCCS Hospital “Giovanni Paolo II” in Bari.

### 3.2. Master and Slave Headsets in the Operating Room

**Figure 3:**
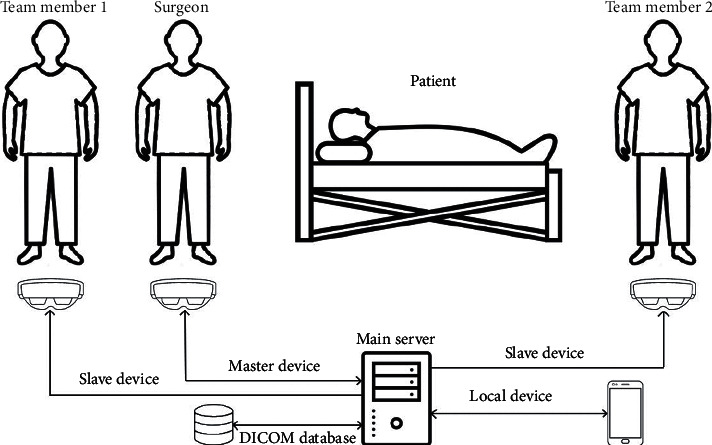
The architecture used for this study.

Figure 3 shows a typical testing scenario in which a surgical team is wearing Microsoft HoloLens. The headset used by the surgeon is marked as the “master” device, and it is directly connected to the main server through a dedicated Wi-Fi connection, whereas the headsets used by the other team members are marked as “slave.”

**Figure 4:**
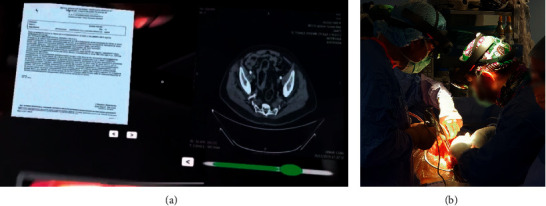
The functionality of the implemented DICOM viewer: (a) the surgeon using the headset to retrieve the content information and (b) the result generated by the viewer application.

The master headset is characterized by a bidirectional connection to the main server. It runs the Spectator View application and shares the information with all the local devices connected to the main server, in order to synchronize them with the same contents generated by the surgeon. All the team members can access all the DICOM information stored in the main database on a nonexclusive basis at the same time. They can use the implemented DICOM viewer application that runs on their devices to ask for recent images. Both the DICOM database and all the local devices support a bidirectional connection with the main server. Namely, when requested by one of the team members, each local device can store a new image into the DICOM database and make it available through the DICOM viewer application. The architecture reported in Figure 3, involving master and slave Microsoft HoloLens headsets, has been adopted in all the ten open abdomen surgeries conducted at the IRCCS Hospital “Giovanni Paolo II.” As an example, Figure 4 shows the case where Microsoft HoloLens has been used during open surgery for right adrenal neoformation exeresis and homolateral nephrectomy with suspected inferior vena cava infiltration. The multidisciplinary approach has aimed at managing high bleeding risk. In this situation, the surgeon wearing the master device was able to visualize radiological images for a better intraoperative finding without having to leave the operating table to view images at any time and to maintain sterility. In the aforementioned figure, it is possible to see the surgeon wearing the master device while scrolling the content retrieved from the DICOM database by using hand gesture to resize the content and scroll through it. It is worth noting that in addition to the images, also text, video, and notes can be overlaid on the real scene. In this scenario, the maximum frame rate used for the HoloLens was 15 fps, with a maximum measured time delay of 125 ms between the master server and all the other devices.

## 4. Discussion on the Microsoft HoloLens for Open Surgery Procedures

As mentioned before, the experimental setup described in the previous section has been used for 10 open abdomen procedures, where at least two Microsoft HoloLens headsets have been used to support the surgical team. After each session, feedback questionnaires have been completed by each team member using Likert scales and by rating their tiredness and fatigue from 1 to 5. The results showed benefits and limits of the use of the Microsoft HoloLens for open surgery procedures.

Referring to the surgical procedures, note that a total of 20 surgical procedures have been performed at the IRCCS Hospital “Giovanni Paolo II,” from March 2019 to February 2020, of which 10 (over 20) have been performed using HoloLens headsets. The main surgical team that took part in all the procedures was composed of the Director of General Medicine Unit and five medical assistants. The average age of the surgeons was 45. The 20 cancer patients treated during all the procedures have been examined by a multidisciplinary team. All the team members strongly suggested surgery in all the 20 cases.

All the 10 surgical procedures based on the use of the Microsoft HoloLens have been related to surgical oncology: two resections of hepatic segments, one metastatic neoplasm on right adrenal gland, two total gastrectomy, and five pancreaticoduodenectomies. The other 10 surgical procedures performed without the use of the HoloLens have been almost the same except for metastases on the left adrenal gland.

### 4.1. Advantages

#### 4.1.1. Mixed Reality and Video Recording for Training

One of the most useful aspect in using the Microsoft HoloLens devices in combination with the implemented applications is the possibility to use the mixed reality functions. Namely, the surgeon with the master device can place useful information on the patient's body to help other members to keep notes of the procedures. In particular, the surgeon can add virtual 3D objects as well as images, text, or video, which can be of guidance during the procedure. Furthermore, the mixed reality function can be used to improve the current surgical training and education system. Namely, efficiency problems are encountered up to 30% of graduating general surgery students, given that most of them are unable to operate independently. By virtue of the mixed reality applications, the surgeon can add visual information and notes to the real scene and to the real body of the patient and share them with external students connected to the main server via their local devices. Furthermore, the video recording feature, available on each Microsoft HoloLens, can be used both as a digital operative report and informational resources for patients about to undergo the same surgery. Video recording might be also used as a coaching video, along with the surgeon's narration.

Another advantage is that during the surgical procedures performed at the IRCCS Hospital, it has been possible to anchor some useful tooltips both for telementoring and medical training. This capability has assured a better identification of anatomical variants and lymph nodes. Moreover, it has highlighted single steps during each surgery, thus improving the educational effectiveness of telementoring programs, given that students can closely follow what happens on the operating table.

#### 4.1.2. DICOM Viewer

**Figure 5:**
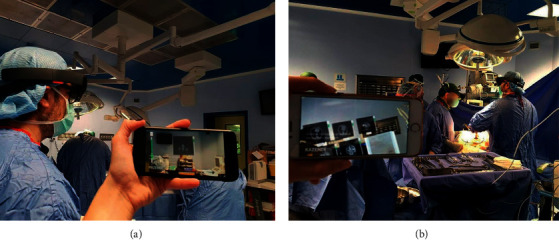
DICOM viewer running on two different local devices: (a) the surgeon with the master device is visualizing information from DICOM viewer and (b) the same information is superimposed on the local device screens.

The developed DICOM Viewer can load medical data in seconds and make them available to the surgeon and the medical team in real time. The capability to load several DICOM data into the database, to convert their content into 3D models, and to load them onto the HoloLens device is a very useful feature. Namely, the surgeon can anchor these kind of objects to points of space within the environment. Additionally, he can rotate or resize the objects, depending on his needs, making them available for the whole team as well as for the local devices.  Figure 5 reports an experimental case, where the surgeon wearing the Microsoft HoloLens configured as master device is visualizing DICOM content while the same information is superimposed over the real scene on local devices. It is worth noting that the virtual content is displayed on the local devices in the same position as placed by the surgeon in the real three-dimensional place. In this scenario, the use of Microsoft HoloLens allows each team member to view and compare CT-MRI-PET/CT images simultaneously during surgery. Moreover, the surgeon has the option of placing the holograms at different points in space for more readability.

### 4.2. Drawbacks

#### 4.2.1. Ergonomicity

**Figure 6:**
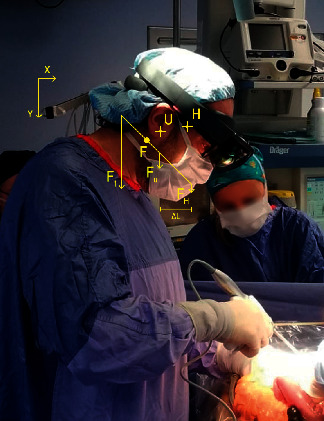
The effects of the headset on the momentum of the gravity force on the surgeon's neck.

The Microsoft HoloLens headset used for this research work is equipped with a head-mounted display worn on the head as image display monitor. Even though wearing the Microsoft HoloLens can improve the performance of each surgical team member, there are some concerns about the drawbacks associated with the weight and the balance of this device. During this study, it has been noted that as the surgery time increases, the physicians were forced to change their neck posture. This leads to a deviation from a neutral upright position. Unfortunately, this incorrect neck posture over a time period can increase the stresses on the musculoskeletal system of the head and neck, thus increasing the potential for injury. As stated by previous studies [24], the weight of the headset may affect the physical workload of wearers because of increased momentum of inertia of the neck and lumbar joints [25]. While wearing the Microsoft HoloLens, the center of mass of the user's head changes because of the weight and the geometrical shape of the device itself. Adding an extra mass to the head can force the user to move away from the natural neck position towards unacceptable regions, leading to a potential damage.In order to put less strain on the user's neck, it is critical to make the vertical line through the center of mass of the headset as close as possible to the vertical line through the center of mass of the height or (if it is not possible) at least slightly behind it. During everyday activities, like standing or sitting, the center of mass of the head is located in front of the atlantoaxial articulation by virtue of the momentum of force generated by the cervical spine extensors. In the human body, the atlantoaxial joint allows complex movements of the cervical spine and provide sufficient mechanical strength to stabilize the head. By wearing the Microsoft HoloLens (that has a weight of about 579 g) and all the hardware located very close to the optical lenses, it is easy to note that an additional momentum of force due to gravity must be considered. This is because the center of mass of the device is anterior to the center of mass of the head.  Figure 6 illustrates the effect of the helmet on the momentum of gravity force related to the atlantoaxial articulation where *U* is the center of mass of the head, *H* is the center of mass of the system composed of the head plus the headset, Δ*L* is the difference in the momentum arm, and *F*_1_ is the force employed by the cervical spine extensor to counterbalance *F*_*H*_ which is the contribution of the center of mass of the surgeon's head and the center of mass of the HoloLens. The wider the module of Δ*L*, the bigger the stress on the extensors.

#### 4.2.2. Reduced Field of View and Optics

**Figure 7:**
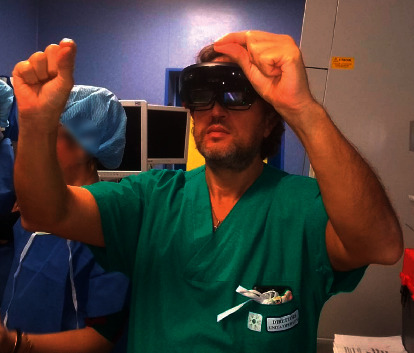
Microsoft HoloLens blocks almost 60% of the total light coming from the environment: the wearer's eyes are half-obscured by the lenses.

Unlike some other mixed reality headsets that are equipped with glass lenses and LED/OLED displays placed right in front of the eyes of the user, HoloLens is a pass-through device. This means that the user is able to see the real world through the device's clear lenses, while the holograms are just projected out in front of him. The optical solution adopted by HoloLens is based on waveguides, which are physical structures in optics able to guide the light wave toward the user's eye. The device adopts a series of diffraction gratings to force the light to enter and exit the waveguide. In particular, there are three waveguides in total: one for the green color, another one for blue color, and a third one for the red color. These diffractive waveguides block about the 60% of the total light coming from the real-world environment and can reach up to 320 cd/m^2^ at full brightness. This is shown in Figure 7, where it is possible to see the eyes and the glasses of the surgeon, which are both darkened.

Moreover, even though the HoloLens has a two-dimensional interpupillary distance (IPD) system able to adjust the eye box horizontally and vertically to fit the distance between the pupils of the subject wearing the headset, their field of view of about 30 × 17.5 degrees is still too much limited and narrow if compared to the average human field of view, which is about 200 degrees for the horizontal view and about 130 degrees for the vertical view. This limitation is caused by two technical reasons. The first is the poor graphics processing power provided by the HoloLens hardware. The second reason is the optical limitation due to the holographic waveguides used to redirect imagery from the left and right display systems into the user's left and right eye views. This kind of waveguides cannot bend incoming light by arbitrary angles, so the maximum field of view obtainable by using holographic waveguides is strictly related to the index of refraction of the waveguide's material, resulting in a narrow field of view. During our research study, we noticed that all the team members were negatively affected by the narrow field of view of the headset, since they were not able to see all virtual objects at all times. In order to compensate, team members were forced to move a lot around their head, thus resulting in an increased stress and fatigue. A feeling of disorientation was perceived every time the members changed the head orientation, after having placed the virtual objects in the three-dimensional environment. Namely, they tried to look back, since they were not able to find again the objects in an easy way. This happens because by using a see-through technology for the headset, virtual objects are cut off at the edges of the display area and simply disappear, even if they are not occluded by anything else. This drawback has been highlighted by all the team members in all the ten open abdomen surgeries conducted at the IRCCS Hospital “Giovanni Paolo II.” It can be concluded that, unfortunately, this limitation makes the HoloLens to be unusable in most situations, even for experienced users. Namely, the surgeons need to use small virtual objects in order to avoid objects suddenly disappearing when hitting the device edges. Thus, the users need to place the objects in their usual field of view. Consequently, this leads to scale down too much useful virtual information, like DICOM images or text notes, with the drawback that images or text becomes not readable.

#### 4.2.3. Parallax Error

**Figure 8:**
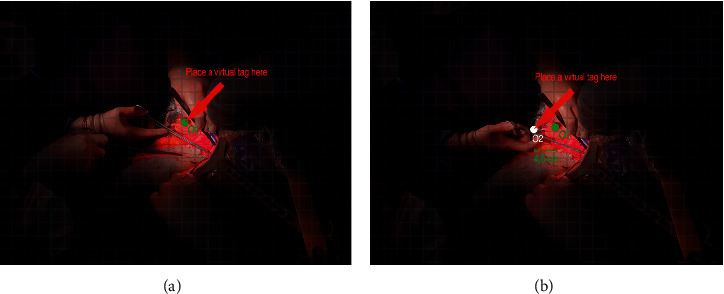
The parallax error might mislead the surgeon about the exact position of a virtual object, thus resulting in serious problems.

Due to optical constraints, parallax errors are very hard to be avoided [26]. This drawback has been reported by the surgeon in all the ten open abdomen surgeries conducted at the IRCCS Hospital “Giovanni Paolo II.” Namely, when the surgeon was operating on a patient that was 50 cm far from his eyes or even closer and he placed a virtual tag at a specific distance, the two virtual images did not match [27]. An example of this drawback is reported in Figure 8, where a virtual tooltip appears to be in two different positions when observed from two different angles. During our study, the parallax error has been empirically evaluated by applying a 20 × 27 square grid on the recorded screen and by calculating the distance between the initial anchor point and all the other observed points related to different view angles. The example reported in Figure 8 shows a displacement between *O*1, which is the first anchor point, and *O*2, which is the same point observed from a different angle. This displacement is about 1.5 squares, which results in a displacement error of about 4.5 centimeters, since each square is about 2 cm. Note that displacement errors can be extremely dangerous, since a surgeon could cut a vessel based on a wrong observation, given that he is viewing the virtual annotation from a different angle. However, even if the overlaid data (i.e., the visual tooltips) are placed by using spatial anchors to keep track of their coordinates over time, they cannot correctly reposition if the patient body is moved or somehow altered during the surgery procedure. This happens because it is not possible to put steady markers directly on the patient's body, given that tissues can be removed, stretched, or repositioned during a complex surgery procedure. This kind of error is difficult to be avoided, since it requires instantaneous tracking of the eyes as well as the understanding of where the surgeon is focusing on.

#### 4.2.4. Headset Autonomy

Microsoft HoloLens is a stand-alone headset that integrates a passively cooled CPU, a Holographic Processing Unit, RAM, and flash memory in a very narrow space with a light battery weight. Obviously, this causes a limited amount of available memory and calculation power available for extra user applications. Moreover, forcing the operating system to run additional applications, like DICOM viewer and other mixed reality utilities, results in increased power consumption that drastically reduces the battery autonomy to a maximum of 2 hours in best cases. Surely, this is another problem to be faced in the operating rooms, particularly when the surgical team has to deal with long surgery procedures.

## 5. Results

Procedural times, physical stress, ease of use, and comfort have been registered and collected during twenty surgical procedures by using survey questionnaire based on Likert scale and operating room records. For ten procedures, team members used standard monitors, tools, and instruments by directly consulting the results of X-ray imaging, radiological CT, PET/TAC, and MRI exams. On the other hand, for the other ten surgical procedures, team members wore Microsoft HoloLens. Each team member had at least 20 hours of experience in the use of mixed reality headsets. Note that the HoloLens has been used for about 2 hours in each surgery (owing to their autonomy), whereas surgical procedures had a maximum duration of about 5 hours.

Table 1 shows the average score based on the sum of each member's feedback following a Likert-type five-point scale where 1 is related to a negative experience and 5 is the maximum degree of satisfaction. It can be seen that the use of Microsoft HoloLens allows the team members to be multitasking, since they can retrieve information without looking away from the patient's body. Moreover, Microsoft HoloLens provides higher image quality with respect to the one achieved by using standard monitors. These features lead to an increased procedural speed. On the other side, physical stress, especially on the neck, is greater when wearing the headset. As described in Figure 4 and as reported by the team members, the amount of stress on the neck is caused by the position usually assumed by the surgeon, which is about 45 degrees towards the patient's body. The stress is also due to the continuous head rotations and movements, which are made in order to retrieve the virtual content owing to the reduced HoloLens field of view. In addition, the surgical team reported a feeling of pressure near the forehead point (*H*), where the greater weight of the visor helmet is applied. During all the ten surgical procedures, the limited reduced field of view forced almost each team member to frequently change his head orientation in order to correctly see over the operating table. This is because the real field of view was obstructed by the HoloLens frame during the surgeries. Furthermore, during the ten interventions with HoloLens visor, the need to change the orientation of the head to be able to frame the operating field (the viewer obstructs the real vision of the field) was demonstrated. It is worth noting that each team member must wear the headset starting from the preoperative phase, when they perform the antisepsis procedure and wear surgical gloves and scrubs. Thus, the total wearing time for the headset is much longer than the duration of the surgical procedure itself. On the one hand, the prolonged use of the headset can cause stress on the neck; on the other hand, it affects the battery lifetime. The practicality of HoloLens is low because of its battery autonomy. Note that four of the ten surgical procedures lasted for more than 3 hours, thus the members had to continue the operation without the headset. Moreover, this study highlighted that the surgeon takes advantage of the use of the headset specifically during the surgical demolition phase, which can last from about 1 hour up to 3 hours, depending on the surgical procedure type. Therefore, the battery life cannot be enough for this task. Unfortunately, this leads to the situation of not being able to use the headset when it is needed the most.

Another downside related to the structure of the HoloLens headset is that it is very uncomfortable if it needs to be used as scene recorder, i.e., for training or academic purposes. This is because the video camera is placed above the lenses, so the surgeon needs to excessively lower his head to improve the camera pictures. Another drawback is that surgeon and his assistant wearing the visor during each intervention always interacted with the virtual content by using gestures. Even though during the first two interventions, some attempts have been made to use the voice interaction, this functionality did not work as expected since it was not very reactive. Consequently, the team has continued to use only gestures in the remaining interventions. The average opinion score of each team member about the use of Microsoft HoloLens for surgical operations is reported in Table 2.

The survey reports how the users are satisfied by the HoloLens performances during the procedures. The survey confirms that the implemented Spectator View can be a valid support in surgical education, too. The use of the developed DICOM Viewer seems to be able to speed up the activities, mainly because it provides the surgeon with high-quality images and real-time patient's information. At the same time, the battery autonomy and the weight of the device are currently considered as serious limitations.

## 6. Conclusion

This paper has exploited a mixed reality platform with the aim to support surgeons (in the operating rooms) during open abdomen surgical procedures. The collected data have clearly shown that the use of Microsoft HoloLens can improve the comfort and can speed up the surgical procedure. Moreover, the proposed DICOM viewer application has proved to effectively support the surgeon to readily retrieve the results of medical screenings, without the need to ask for additional aid. Furthermore, the integration of the Spectator View tool has enabled any data to be shared by the team members or by external users during surgical operations, this being a remarkable feature for supporting education and training methods. These advantages have been encountered by surgeons during all the ten open abdomen surgeries conducted at the IRCCS Hospital “Giovanni Paolo II” in the city of Bari. The paper has also analysed in detail some drawbacks, such as the low autonomy in the battery of the headset and the physical stress, which can increase the potential for a neck injury due to incorrect posture during the surgery procedure. A survey based on Likert scale has been reported, which has clearly demonstrated how the use of the suggested tools can increase the execution speed by allowing multitasking procedures, i.e., by checking medical images at high resolution without leaving the operating table and the patient. Additionally, the outcome of the survey has shown that the use of both DICOM Viewer and Spectator View is useful for surgical education purposes. We would highlight that the real use of the conceived platform in the operating room has represented a remarkable feature of our approach, since most if not all studies conducted so far in literature exploit mixed reality only in simulated environments [28] and not in real operating rooms. In conclusion, the authors believe that mixed reality represents a promising technique that in a few years will enter the operating rooms to support surgeons during surgical procedures in many hospitals across the world. However, some technological improvements are required, since current drawbacks prevent medical staff to adopt the available devices as standard procedures in the operating rooms. These improvements involve headsets with a more weight-balanced structure, high-quality cameras, and a battery with longer autonomy.

## Figures and Tables

**Table 1:** User feedback and differences with (*H*) and without (SM) Microsoft HoloLens.

	*H*	SM
Execution speed	4	3
Physical stress	5	3
Comfort	3	4
Practicality	2	4
Multitasking	5	3
Image quality	5	4

**Table 2:** Opinions after the use of Microsoft HoloLens.

	1–5
The use of HoloLens is useful in surgical procedures	4
DICOM Viewer is useful to speed up the procedure	5
HoloLens can improve surgical operations	4
The use of HoloLens and Spectator View can improve surgical education	5
Battery autonomy should be improved	5
The weight of the device is not relevant	1

## Data Availability

The data that support the findings of this study are available from the corresponding author upon request.
